# GP120 and tenofovir alafenamide alter cannabinoid receptor 1 expression in hippocampus of mice

**DOI:** 10.1007/s13365-023-01155-x

**Published:** 2023-10-06

**Authors:** Jacqueline Renee Kulbe, Alexandra Anh Le, Michael Mante, Jazmin Florio, Anna Elizabeth Laird, Mary K. Swinton, Robert A. Rissman, Jerel Adam Fields

**Affiliations:** 1 University of California, San Diego Department of Psychiatry, San Diego, CA USA; 2 University of California, San Diego Department of Neurosciences, San Diego, CA USA; 3 Department of Physiology and Neuroscience, Keck School of Medicine of USC, Alzheimer’s Therapeutic Research Institute, San Diego, CA USA

**Keywords:** HIV, Astrogliosis, Endocannabinoid, TAF, gp120

## Abstract

Central nervous system (CNS) dysfunction remains prevalent in people with HIV (PWH) despite effective antiretroviral therapy (ART). There is evidence that low-level HIV infection and ART drugs may contribute to CNS damage in the brain of PWH with suppressed viral loads. As cannabis is used at a higher rate in PWH compared to the general population, there is interest in understanding how HIV proteins and ART drugs interact with the endocannabinoid system (ECS) and inflammation in the CNS. Therefore, we investigated the effects of the HIV envelope protein gp120 and tenofovir alafenamide (TAF) on cannabinoid receptor 1 (CB_1_R), glial fibrillary acidic protein (GFAP), and IBA1 in the brain and on locomotor activity in mice. The gp120 transgenic (tg) mouse model was administered TAF daily for 30 days and then analyzed using the open field test before being euthanized, and their brains were analyzed for CB_1_R, GFAP, and IBA1 expression using immunohistochemical approaches. CB_1_R expression levels were significantly increased in CA1, CA2/3, and dentate gyrus of gp120tg mice compared to wt littermates; TAF reversed these effects. As expected, TAF showed a medium effect of enhancing GFAP in the frontal cortex of gp120tg mice in the frontal cortex. TAF had minimal effect on IBA1 signal. TAF showed medium to large effects on fine movements, rearing, total activity, total distance, and lateral activity in the open-field test. These findings suggest that TAF may reverse gp120-induced effects on CB_1_R expression and, unlike tenofovir disoproxil fumarate (TDF), may not affect gliosis in the brain.

## Introduction

Nearly 40 million people worldwide, including 2 million people within the United States, are infected with HIV. Antiretroviral therapies (ART) decrease viral loads, prolong life expectancy, and reduce overall morbidity in people living with HIV (PWH). However, approximately 50% of PWH on ART will develop an HIV-associated neurocognitive disorder (HAND) or another central nervous system (CNS) disorder such as depression (Heaton et al. 2011). HAND represents a spectrum of severities including asymptomatic neurocognitive impairment (ANI), mild neurocognitive impairment (MCI), and HIV-associated dementia (HAD) (Cotto et al. 2019b). Although ART has decreased the incidence of HAD (Saylor et al. 2016), PWH still experience premature cognitive impairment compared to the general population and despite suppressed viral loads can demonstrate impairments to executive function, memory, and motor skills (Cotto et al. 2019b). Similar to other neurocognitive disorders, HAND leads to decreased quality of life, increased healthcare costs, and increased caregiver burden. Despite much effort, therapies against HAND are not available and novel mechanistic targets are needed for this susceptible population.

The pathogenesis of HAND in PWH on ART is likely multifactorial and includes factors such as an aging population, damage to the blood-brain-barrier (BBB), CNS toxicity by the ART drugs that cross the BBB and continued low-level HIV protein expression from the CNS viral reservoir leading to persistent neuroinflammation and metabolic dysfunction (Castellano et al. 2019; Donoso et al. 2022; Ferrara et al. 2020; Harezlak et al. 2011) (Cotto et al. 2019a, b; Fields et al. 2016b; Garvey et al. 2014). Astrogliosis, pro-inflammatory microglia, and neuronal damage persist in the brains of PWH on ART (Fields et al. 2018; Swinton et al. 2019). Our previous study using the gp120 tg mouse model revealed that tenofovir disoproxil fumarate (TDF) increases astrogliosis while reducing microgliosis in the brain (Fields et al. 2019). Tenofovir alafenamide (TAF) is the 2^nd^ tenofovir prodrug; it is capable of crossing the blood brain barrier (Gele et al. 2021), and it was developed to reduce renal toxicity (Di Perri 2021). The effects of TAF on the brain in gp120tg mice have yet to be investigated.

We and others reported that neuroinflammation PWH is concomitant with changes in endocannabinoid system gene expression including cannabinoid receptor 1 (CB_1_R) and cannabinoid receptor 2 (CB_2_R) (Cosenza-Nashat et al. 2011; Swinton et al. 2021). This may be important because cannabis use is prevalent in PWH. Moreover, multiple studies have indicated that CBR agonists are neuroprotective against HIV- and specifically gp120-induced neurotoxicity and in PWH (Avraham et al. 2011; Ellis et al. 2021b; Swinton et al. 2019; Watson et al. 2021). Despite these findings, we are unaware of any studies investigating CBR expression in a mouse model's brains expressing the toxic gp120.

Envelope glycoprotein 120 is a neurotoxic HIV protein that when expressed in the brains of mice recapitulates much of HAND pathology including astrogliosis, microgliosis, mitochondrial alterations, and memory impairment (Avdoshina et al. 2016; Fields et al. 2016a, b; Toggas et al. 1994). Because the purpose of this study was to investigate the effects of TAF in an animal model of HAND, 12-month old mice were used. Previous studies have demonstrated that at 12-months of age gp120 mice have loss of neuronal dendrites and synapses, increases in microgliosis (Iba1), astrogliosis (GFAP), reduced swimming velocity, impaired spatial retention memory, alterations in open field and activity measures, increases in peripheral neuropathy, but no significant impairment in motor function as measured by the rotarod test (D'Hooge et al. 1999; Thaney et al. 2018).

Additionally, gp120 transgenic mice may represent a relevant model for HIV infection in the ART era because although ART is able to suppress HIV viral replication, viral proteins such as gp120 can remain. Additionally, our previous study demonstrated that although TDF attenuates gp120-induced increases in the pro-inflammatory cytokine TNFα in a microglial cell line, it also significantly increased glial fibrillary acidic protein (GFAP) expression and TNFα mRNA levels in wild-type mice (Fields et al. 2019), which may have concerning implications for its long-term use as an ART drug.

In this follow-up study, we evaluated the effects of TAF on cortex and hippocampal expression of GFAP, IBA1, and CB_1_R and locomotor activity in wild-type and gp120tg mice. This is the first study to examine the effects of TAF on astrogliosis, microgliosis, CB_1_R expression, and locomotor activity in gp120 and wild-type mice. Our hypothesis was that TAF would attenuate gp120-induced increases in GFAP, IBA1, and CB_1_R expression, improve gp120-induced locomotor deficits, and that contrary to TDF, TAF would not be pro-inflammatory in wild-type mice. In line with our hypothesis, GP120 increased CB_1_R expression in mouse hippocampi, which is reversed by TAF treatment, TAF treatment had a moderate effect on locomotor activity, and TAF did not increase GFAP expression in wild-type mice. However, contrary to our hypothesis TAF treatment had no effect on GFAP expression in gp120-tg or wild-type mice and there was a limited effect on IBA1.

## Methods

### Animals

For these studies, we utilized an animal model of HIV-1-protein-mediated neurotoxicity, which expresses high levels of gp120 under the control of the GFAP promoter (Toggas et al. 1994). These mice develop neurodegeneration accompanied by astrogliosis, microgliosis, and behavioral deficits (Toggas et al. 1996). As previously described (Crews et al. 2010), 9-month-old non-tg and gp120 tg animals (total of 40 mice; *n* = 10 per group; *n* = 5 male and *n* = 5 female mice/group) received daily intraperitoneal (IP) injections with saline (vehicle) alone or TAF (MedChemExpress, cat. no. HY-15232) at a concentration of 50 mg/kg for 4 weeks. The mice were sacrificed following locomotor testing, and brains were removed and fixed brain tissues before being processed with a vibratome to generate 40 µm free-floating sections.

Our previous study evaluating TDF in gp120 mice used TDF at 50mg/kg by IP for four weeks which through allometric scaling equates to 42 mg/kg/day or 29 mg/70 kg person (Fields et al. 2019), falling well below the standard clinical dose of 300mg/day. However, is quite concerning given its ability to induce peripheral neuropathy and inflammation (Fields et al. 2019) at a dose far below what is clinically prescribed. In this study the same dosing paradigm was chosen for TAF, a pro-drug of TDF that produces higher plasma levels of active drug, allowing it to be dosed in smaller quantities. The clinical dose for TAF is 25mg/day, therefore the 29mg/70kg person conversation remains clinically relevant. Although, for persons that greatly exceed 70kg would represent a supratherapeutic dose.

### Locomotor Activity Assessment

The open-field locomotor test was used to determine basal activity levels of study subjects (total move time) during a 15-min session. Spontaneous activity in an open field (25.5 × 25.5 cm) was monitored for 15 min using an automated system (Truscan system for mice; Coulbourn Instruments, Allentown, PA). Animals were tested within the first 2–4 hours of the dark cycle after being habituated to the testing room for 15 min. The open field was illuminated with an anglepoise lamp equipped with a 25-W red bulb. Animals were tested at 12 months of age (after 4 weeks of treatment). Time spent in motion was automatically collected 3 × 5 min time bins using the TruScan software. Data were analyzed for both the entire 15-min session and for each of the 5-min time blocks.

### Immunohistochemistry of brain sections

As previously described (Fields et al. 2019), free-floating 40 μm thick vibratome sections of mouse brains were washed with phosphate-buffered saline with tween 20 (PBST) 3 times, pre-treated for 20 minutes in PBS 3% H2O2/1%TritonX, and blocked with 2.5% horse serum (Vector Laboratories) for 1 hour at room temperature. Sections were incubated at 4 C overnight with the primary antibodies, CB_1_R (Abcam, cat. no. ab23703) GFAP (Sigma, cat. No. G3893), and IBA1 (Wako, cat. no. 019–19741). Sections were then incubated in a secondary antibody, Immpress HRP Anti-rabbit IgG (Vector, cat. no. MP-7401) or Impress HRP Anti-mouse IgG (Vector, cat. No. MP-7402) for 30 minutes, followed by NovaRED Peroxidase (HRP) Substrate made with NovaRED Peroxidase (HRP) Substrate Kit as per manufacturer’s instructions (Vector, cat. no. SK-4800). Control experiments consisted of incubation with secondary antibodies only. Tissues were mounted on Superfrost plus slides and coverslipped with cytoseal. Immunostained sections were imaged with a digital Olympus microscope and assessment of levels of CB_1_R, GFAP, and IBA1 immunoreactivity was performed utilizing the Image-Pro Plus program (Media Cybernetics, Silver Spring, MD). For each case, each area of interest was analyzed in order to estimate the average intensity of the immunostaining (corrected optical density). Background levels were obtained in tissue sections immunostained in the absence of primary antibody. Therefore: corrected optical density = optical density − background. The intensity of GFAP and IBA1 positive cells were quantified in the hippocampi using ImageScope analysis software. Areas analyzed included the frontal cortex, and the following areas of the hippocampus; CA1, CA2/3, and DG.

### Statistical Analysis

Statistical analysis included two-way ANOVA on corrected optical densities from immunostainings. Additionally, Cohen's *d* and effect sizes were calculated between treatment groups (Tables 1-4). The sample size (*n*) of CB_1_R, GFAP, and IBA1 are as follows and listed in this order: wt-vehicle, wt-TAF, gp120-tg vehicle, gp120-tg TAF. For CB_1_R–CA1 (n = 7, 8, 9, 11), CB_1_R–CA2/3 (n = 8, 8, 9, 9 ), CB_1_R–DG (n = 7, 8, 9, 10), CB_1_R–FC (n = 7, 8, 9, 11). For GFAP–CA1 (n = 10, 9, 10, 10), GFAP–CA2/3 (n = 10, 9, 10, 10), GFAP–DG (n = 10, 9, 10, 10), GFAP–FC (n = 9, 9, 10, 10). For IBA1–CA1 (n = 10, 10, 9, 10), IBA1–CA2/3 (n = 10, 9, 9, 9), IBA1–DG (n = 10, 10, 9, 9 ), IBA1–FC (n = 10, 10, 10, 10). In the same order, the sample size for locomotor activity assessments are as follows: fine movements (n = 9, 10, 10, 10), total activity (n = 9, 10, 10, 10), lateral activity (n = 10, 10, 10, 10), total distance (n = 9, 10, 10, 10), rearing (n = 9, 10, 10, 10), time in periphery (n = 10, 10, 10, 10), and thigmotaxis (n = 10, 10, 10, 10). The n/group vary between experiments due to outliers being removed, which were identified as being two standard deviations outside the mean for that group.

## Results

**Fig. 1:**
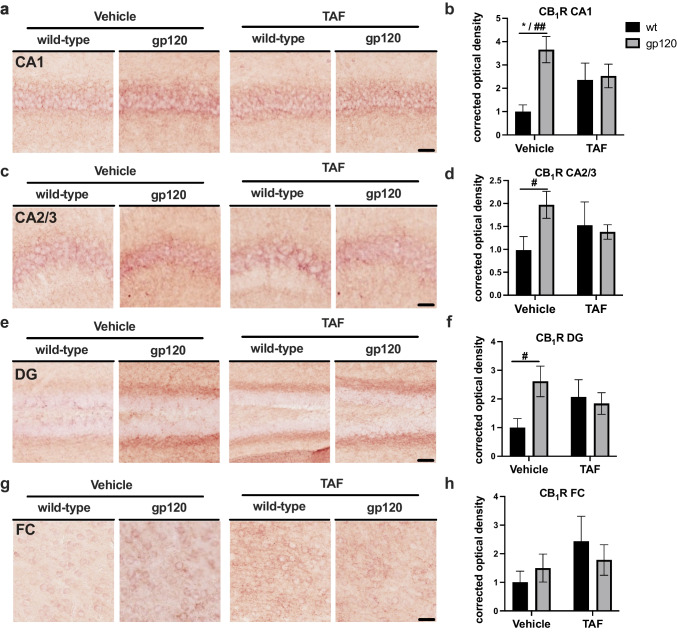
GP120 increases CB_1_R expression in mouse hippocampi, which is reversed by TAF treatment** a **CB_1_R immunostaining of CA1. **b** Quantification of corrected optical density for CA1. **c** CB_1_R immunostaining of CA2/3. **d** Quantification of corrected optical density for CA2/3. **e** CB_1_R immunostaining of DG. **f** Quantification of corrected optical density for DG. **g** CB_1_R immunostaining of FC. **h** Quantification of corrected optical density for FC. Analyzed with two-way ANOVA (*) and T-tests (#): *p,0.05, ^#^*p*<0.05, ^##^*p*<0.01

**GP120 increases CB**_**1**_**R expression in mouse hippocampi, which is reversed by TAF treatment.** To determine if TAF influences CB_1_R expression in wt and gp120-tg mice, we immunostained vibratome sections for CB_1_R and performed intensity/area analysis. Areas analyzed included the following regions of the hippocampus (HC); CA1, CA2/3, and DG and the frontal cortex (FC). In the CA1 region of the HC, CB_1_R signal was more intense in gp120-tg mice compared to wt in vehicle-treated mice (Fig. 1a). However, CB_1_R signal in CA1 appeared similar in gp120-tg and wt mice treated with TAF, with the signal in both being less intense than in vehicle-treated gp120-tg mice (Fig. 1a). Quantification of corrected optical density in the CA1 region revealed a ~250% increase in CB_1_R signal in gp120-tg versus wt mice (*p*<0.01; cohen’s d = 2.03; Fig. 1b). Interestingly, TAF reduced the CB_1_R signal by ~30% in the gp120-tg mice compared to vehicle-treated gp120-tg mice (Cohen’s d = 0.67) and increased the CB_1_R signal in TAF-treated wt compared to vehicle treated wt mice (Cohen’s d = 0.88; Fig. 1b**)**. In the CA2/3 region of the HC, CB_1_R signal was more intense in gp120-tg mice compared to wt in vehicle-treated mice (Fig. 1c). However, CB_1_R signal in CA2/3 appeared similar in gp120-tg and wt mice treated with TAF, with the signal in both being less intense than in vehicle-treated gp120-tg mice (Fig. 1c). Quantification of corrected optical density in the CA2/3 region revealed a ~100% increase in CB_1_R signal in gp120-tg versus wt mice (*p*<0.05; cohen’s d = 1.15; Fig. 1d). Interestingly and similar to the CA1 region, TAF reduced the CB_1_R signal by ~30% in the gp120-tg mice compared to vehicle-treated gp120-tg mice (Cohen’s d = 0.84) and increased the CB_1_R signal in TAF-treated wt compared to vehicle-treated wt mice (Cohen’s d = 0.46; Fig. 1d**)**. In the DG region of the HC, CB_1_R signal was more intense in gp120-tg mice compared to wt in vehicle-treated mice (Fig. 1e). Again, CB_1_R signal in the DG appeared similar in gp120-tg and wt mice treated with TAF, with the signal in both being less intense than in vehicle-treated gp120-tg mice (Fig. 1e). Quantification of corrected optical density in the DG region revealed a ~160% increase in CB_1_R signal in gp120-tg versus wt mice (*p*<0.05; cohen’s d = 1.26; Fig. 1f). TAF reduced the CB_1_R signal by ~25% in the gp120-tg mice compared to vehicle-treated gp120-tg mice (Cohen’s d = 0.55) and increased by ~100% the CB_1_R signal in TAF-treated wt compared to vehicle-treated wt mice (Cohen’s d = 0.79; Fig. 1f**)**. In the FC, CB_1_R signal was only slightly more intense in gp120-tg mice compared to wt in vehicle-treated mice (Fig. 1g). The CB_1_R signal in FC appeared similar in gp120-tg and wt mice treated with TAF, with the signal in both being more intense than in vehicle-treated gp120-tg and wt mice (Fig. 1g). Quantification of corrected optical density in the FC revealed no significant difference (and only small to medium effect sizes between all groups) in CB_1_R signal in gp120-tg versus wt mice (Fig. 1h). Effect sizes for differences between groups are illustrated in Table 1.

**Table 1:** Cohen’s d and effect size for CB_1_R

	**CA1**		**CA2/3**		**DG**		**FC**	
**Groups**	***d***	**Effect Size**	***d***	**Effect Size**	***d***	**Effect Size**	***d***	**Effect Size**
wt-v vs. wt-TAF	0.88	large	0.46	small	0.79	medium	0.76	medium
wt-v vs. g-v	2.03	large	1.15	large	1.26	large	0.39	small
wt-v vs. g-TAF	1.17	large	0.58	medium	0.81	large	0.54	medium
wt-TAF vs. g-v	0.69	medium	0.37	small	0.33	small	0.46	small
wt-TAF vs. g-TAF	0.09	small	0.14	small	0.15	small	0.30	small
g-v vs. g-TAF	0.67	medium	0.84	large	0.55	medium	0.17	small

### TAF treatment increased GFAP expression in the Frontal Cortex but has a variable effect in Hippocampus of gp120-tg mice

**Fig. 2:**
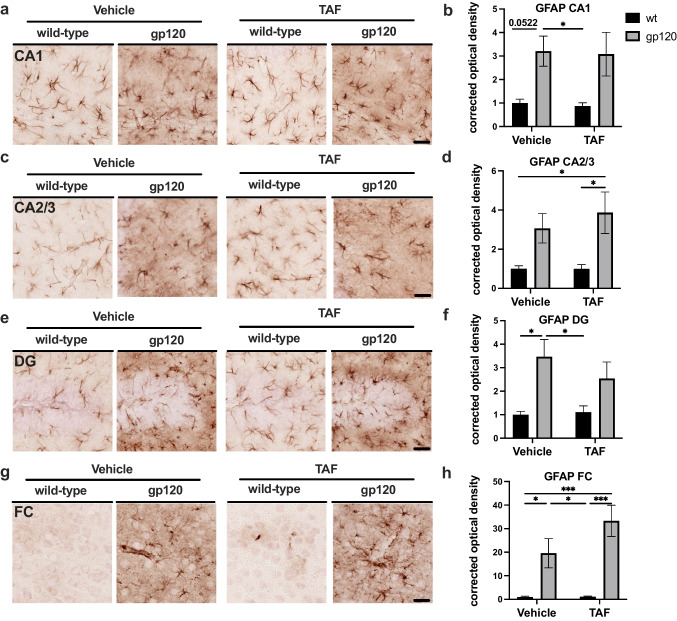
TAF treatment has minimal effect on GFAP expression in the Frontal Cortex and Hippocampus of gp120-tg and wt mice** a** GFAP immunostaining of CA1. **b** Quantification of corrected optical density for CA1. **c** GFAP immunostaining of CA2/3. **d** Quantification of corrected optical density for CA2/3. **e** GFAP immunostaining of DG. **f** Quantification of corrected optical density for DG. **g** GFAP immunostaining of FC. **h** Quantification of corrected optical density for FC. Analyzed with two-way ANOVA: *p<0.05, ***p<0.0001

GFAP is an intermediate filament-III protein found only in astrocytes of the CNS, non-myelinating Schwann cells, and enteric glial cells (Yang and Wang 2015). GFAP expression increases in reactive astrocytes due to the elongation of astroglia processes when compared to their inactivated state (Yang and Wang 2015). Previous studies show that TDF increases GFAP expression in the hippocampus of gp120-tg mice (Fields et al. 2019). To evaluate the status of astroglial activation following treatment with TAF in gp120-tg and wt mice, we immunostained vibratome sections of mouse brains with antibody for GFAP using NovaRed for visualization and analyzed HC and FC. Areas analyzed included the following regions of the hippocampus (HC); CA1, CA2/3, and DG and the frontal cortex. In the CA1 region of the HC, GFAP signal was more intense in gp120-tg mice compared to wt in vehicle- and TAF-treated mice (Fig. 2a). Quantification of corrected optical density in the CA1 region revealed a c200% increase in GFAP signal in gp120-tg versus wt mice in vehicle- and TAF treated mice (*p*<0.05; Fig. 2b). In the CA2/3 region of the HC, GFAP signal was more intense in gp120-tg mice compared to wt in vehicle- and in TAF-treated mice (Fig. 2c). Quantification of corrected optical density in the CA2/3 region revealed a ~200% increase in GFAP signal in gp120-tg versus wt mice (Fig. 2d). In the DG region of the HC, GFAP signal was more intense in gp120-tg mice compared to wt in vehicle- and TAF-treated mice (Fig. 2e). Quantification of corrected optical density in the DG region revealed a ~200% and ~150% increase in GFAP signal in gp120-tg versus wt mice in vehicle- and TAF-treated mice, respectively (Fig. 2f). In the FC, GFAP signal was more intense in gp120-tg mice compared to wt in vehicle- and TAF-treated mice (Fig. 2g). Quantification of corrected optical density in the FC revealed a ~90% increase in GFAP signal between vehicle-treated and TAF-treated gp120-tg mice (Cohen’s d = 0.68) (Fig. 2h). Effect sizes for differences between groups are illustrated in Table 2.

**Table 2:** Cohen's d and effect size for GFAP

	**CA1**		**CA2/3**		**DG**		**FC**	
**Groups**	***d***	**Effect Size**	***d***	**Effect Size**	***d***	**Effect Size**	***d***	**Effect Size**
wt-v vs. wt-TAF	0.17	small	0.01	small	0.16	small	0.10	small
wt-v vs. g-v	1.38	large	1.21	large	1.47	large	1.34	large
wt-v vs. g-TAF	0.95	large	1.20	large	0.95	large	2.18	large
wt-TAF vs. g-v	1.59	large	1.19	large	1.35	large	1.34	large
wt-TAF vs. g-TAF	1.05	large	1.03	large	0.85	large	2.18	large
g-v vs. g-TAF	0.05	small	0.25	small	0.41	small	0.68	medium

### TAF treatment has no effect on IBA1 expression in gp120-tg and wt mice

**Fig. 3:**
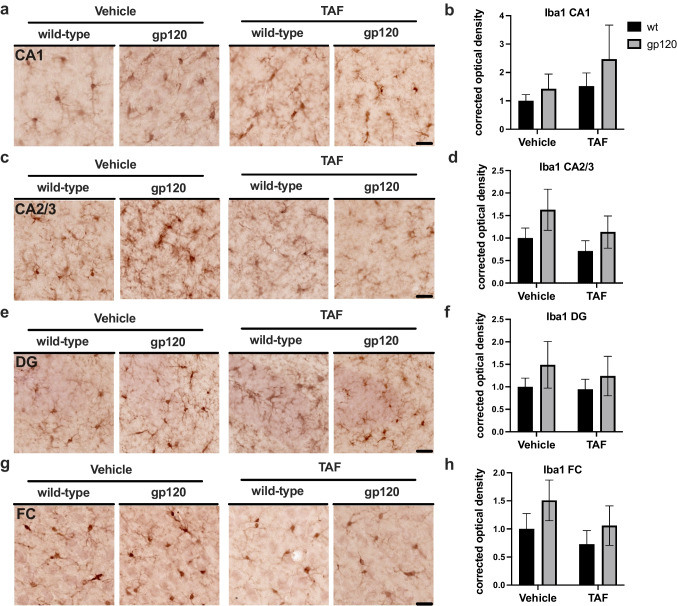
TAF treatment has no effect on IBA1 expression in gp120-tg and wt mice** a** IBA1 immunostaining of CA1. **b** Quantification of corrected optical density for CA1. **c** IBA1 immunostaining of CA2/3. **d** Quantification of corrected optical density for CA2/3. **e** IBA1 immunostaining of DG. **f** Quantification of corrected optical density for DG. **g** IBA1 immunostaining of FC. **h** Quantification of corrected optical density for FC. Analyzed with two-way ANOVA

To evaluate the status of microglial activation following treatment with TAF in gp120-tg and wt mice, we immunostained vibratome sections of mouse brains with antibody for IBA1 using NovaRed for visualization and analyzed HC and FC. Areas analyzed included the following regions of the hippocampus (HC); CA1, CA2/3, and DG and the frontal cortex. In the CA1 region of the HC, IBA1 signal was slightly more intense in gp120-tg mice compared to wt in vehicle- and TAF-treated mice (Fig. 3a), but quantification of corrected optical density in the CA1 region revealed no significnant changes in IBA1 signal between any groups (Fig. 3b). In the CA2/3 region of the HC, IBA1 signal was slightly more intense in gp120-tg mice compared to wt in vehicle- and TAF-treated mice (Fig. 3c), but quantification of corrected optical density in the CA2/3 region revealed no significant changes in IBA1 signal between any groups (Fig. 3d). In the DG region of the HC, IBA1 signal was slightly more intense in gp120-tg mice compared to wt in vehicle- and TAF-treated mice (Fig. 3e), but quantification of corrected optical density in the DG region revealed no significnant changes in IBA1 signal between any groups (Fig. 3f). In the FC, IBA1 signal was slightly more intense in gp120-tg mice compared to wt in vehicle- and TAF-treated mice (Fig. 3g), but quantification of corrected optical density in the FC region revealed no significnant changes in IBA1 signal between any groups (Fig. 3h). Effect sizes for differences between groups are illustrated in Table 3.

**Table 3:** Cohen's d and effect size for IBA1

	**CA1**		**CA2/3**		**DG**		**FC**	
**Groups**	***d***	**Effect Size**	***d***	**Effect Size**	***d***	**Effect Size**	***d***	**Effect Size**
wt-v vs. wt-TAF	0.45	small	0.42	small	0.09	small	0.33	small
wt-v vs. g-v	0.35	small	0.58	medium	0.41	small	0.50	medium
wt-v vs. g-TAF	0.54	medium	0.15	small	0.23	small	0.06	small
wt-TAF vs. g-v	0.06	small	0.84	large	0.45	small	0.80	large
wt-TAF vs. g-TAF	0.33	small	0.47	small	0.28	small	0.34	small
g-v vs. g-TAF	0.36	small	0.40	small	0.17	small	0.40	small

### TAF treatment has a moderate effect on locomotor activity

**Fig. 4:**
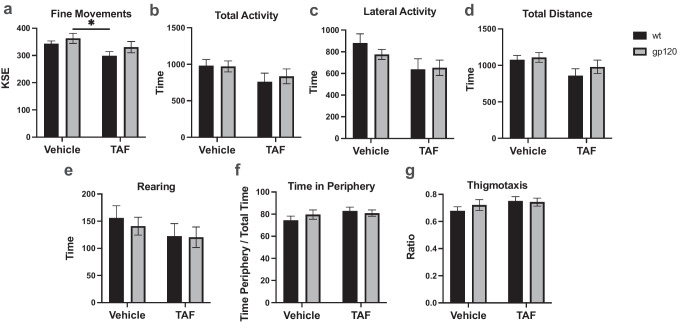
TAF treatment has a moderate effect on locomotor activity. Measurements of **a** fine movements, **b** total activity, **c** lateral activity, **d** total distance, **e** rearing, **f** time in periphery, and **g** thigmotaxis. Analyzed with two-way ANOVA. *p<0.05

To determine if TAF has any effect on locomotor activity, measurements of fine movements, total activity, lateral activity, total distance, rearing, time in periphery, and thigmotaxis were measured in vehicle- and TAF-treated wt and gp120-tg mice. In the wt and gp120-tg groups, treatment with TAF decreased fine movements (Fig. 4a; Cohen’s d = 1.09), total activity (Fig. 4b; Cohen’s d = 0.68), lateral activity (Fig. 4c; Cohen’s d = 0.84), total distance traveled (Fig. 4d; Cohen’s d = 0.88), and rearing (Fig. 4e; Cohen’s d = 0.48). In the wt and gp120-tg groups, treatment with TAF increased time in periphery (Fig. 4f; Cohen’s d = 0.71) and thigmotaxis (Fig. 4g; Cohen’s d = 0.74). Effect sizes for differences between groups are illustrated in Table 4.

**Table 4:** Cohen's d and effect size for locomotor activity

	**Fine Movements**		**Total Activity**		**Lateral Activity**		**Total Distance**		**Rearing**		**Time in Periphery**		**Thigmotaxis**	
**Groups**	***d***	**Effect Size**	***d***	**Effect Size**	***d***	**Effect Size**	***d***	**Effect Size**	***d***	**Effect Size**	***d***	**Effect Size**	***d***	**Effect Size**
wt-v vs. wt-TAF	1.09	large	0.68	medium	0.84	large	0.88	large	0.48	small	0.71	medium	0.74	medium
wt-v vs. g-v	0.42	small	0.04	small	0.50	medium	0.17	small	0.25	small	0.41	small	0.38	small
wt-v vs. g-TAF	0.25	small	0.50	medium	0.95	large	0.40	small	0.56	medium	0.61	medium	0.69	medium
wt-TAF vs. g-v	1.18	large	0.67	medium	0.57	medium	0.97	large	0.29	small	0.25	small	0.26	small
wt-TAF vs. g-TAF	0.55	medium	0.21	small	0.05	small	0.40	small	0.03	small	0.18	small	0.08	small
g-v vs. g-TAF	0.52	medium	0.48	small	0.69	medium	0.51	medium	0.37	small	0.11	small	0.20	small

## Discussion

This study provides the first evidence that TAF, the second generation of tenofovir prodrugs, modulates the ECS in the brain. This study also identifies alterations in CB_1_R in the HC of gp120-tg mice, corroborating previous findings of altered CBRs in postmortem brains of PWH. Finally, we illustrate that TAF reduces multiple measures of locomotor activity in wt and gp120-tg mice. Overall, these findings point to why the ECS may be a targetable system in the brains of PWH.

Despite the increased life expectancy and decreased rates of severe dementia compared to the pre-ART era, HAND remains prevalent in PWH on ART and ongoing neuroinflammation is likely a contributing factor (Cotto et al. 2019b; Garvey et al. 2014; Harezlak et al. 2011). PWH on ART, as well as people at high risk of contracting HIV on PrEP, are chronically exposed to ART drugs, a variety of which have been implicated in neurotoxicity and dysregulation of glia (Fields and Ellis 2019; Fields et al. 2019; George et al. 2021; Tripathi et al. 2020; Xu et al. 2017). However, there remains a dearth of information on their role in neuroinflammation, particularly in regard to newer agents such as TAF. Moreover, compared to the general population, PWH disproportionately consume cannabis and there is some evidence that it may be neuroprotective and anti-inflammatory in this population (Ellis et al. 2021a; Ellis et al. 2020; Pacek et al. 2018; Watson et al. 2021). This is the first study to examine the effects of TAF on astrogliosis, microgliosis, CB_1_R expression, and locomotor activity in gp120-tg and wt mice. Our hypothesis was that TAF would attenuate gp120-induced increases in GFAP, IBA1, and CB_1_R expression, improve gp120-induced locomotor deficits, and that contrary to TDF, TAF would not be pro-inflammatory in wt mice. In line with our hypothesis, GP120 increased CB_1_R expression in mouse HC, which was reversed by TAF treatment, TAF treatment had a moderate effect on locomotor activity, and TAF did not increase GFAP expression in wild-type mice. However, TAF treatment did not attenuate GFAP expression in gp120-tg mice. Although this result was contrary to our hypothesis, it was not entirely unexpected because in a previous study TDF also did not decrease gp120-induced increases in GFAP (Fields et al. 2019).

The endogenous ECS serves a variety of functions, with the predominant receptors being CB_1_R and CB_2_R. CB_1_R receptors are the most abundant g-protein coupled receptors in the brain and stimulation of CB_1_R receptors results in activation of antioxidant defenses (ex: Nrf2 pathway), alterations in glutamatergic and calcium signaling, regulation of neurogenesis and induction of long-term potentiation. CB_1_R receptors are highly expressed in the prefrontal cortex, hippocampus, amygdala, basal ganglia, and cerebellum, inferring importance in cognitive and motor functions (Tadijan et al. 2022). CB_1_R receptors are primarily found in neuronal synaptic terminals but are also expressed in M1 and M2 microglia phenotypes and astroglia (Eraso-Pichot et al. 2023; Tadijan et al. 2022; Young and Denovan-Wright 2021). Additionally, CB_1_R receptors are known to have an anti-inflammatory role in inflammasome formation (Leonard 2023), decrease pro-inflammatory cytokines and increase anti-inflammatory cytokines in the presence of inflammatory stimuli (Walter and Stella 2004), and modulate inflammatory nociception and hyperalgesia (Clayton et al. 2002). On the other hand, CB_2_R receptors are primarily expressed by microglia and are also involved in immune function. Although CB_2_R receptors are consistently expressed at low levels in the healthy CNS, they are upregulated in response to inflammation and immune stimulation (Tadijan et al. 2022; Young and Denovan-Wright 2021). Alterations in CB_1_R and CB_2_R receptor expression has been found in a variety of neurodegenerative disorders (Swinton et al. 2021; Tadijan et al. 2022; Young and Denovan-Wright 2021), and increased CB_1_R expression is found in post-mortem frontal cortex of PWH on ART with minor neurocognitive dysfunction (MND) or HIV-associated dementia (HAD) but not in neurocognitively unimpaired (NUI) or asymptomatic neurocognitive impairment (ANI), with increases in CB_1_R expression being correlated with poorer cognitive function (Swinton et al. 2021). Consistent with previous findings (Cosenza-Nashat et al. 2011; Swinton et al. 2021), our results indicate that CB_1_R expression was significantly increased, with a large effect size, in the HC, but not the FC of gp120 mice, indicating that the HIV gp120 protein is sufficient to induce CB_1_R expression. It is unclear at this time whether increases in hippocampal CB_1_R expression during HIV infection are beneficial or detrimental, and further studies are necessary. However, previous studies have demonstrated that CB_1_R agonists are protective in HAND and gp120-toxicity (Avraham et al. 2011; Ellis et al. 2021b; Lu et al. 2008; Swinton et al. 2019; Watson et al. 2021). Therefore, we propose that increases in CB_1_R expression in gp120 are compensatory and an attempt at neuroprotection, making CB_1_R an attractive pharmacologic target for therapeutic intervention. Several possible upstream mechanisms leading to increased CB_1_R expression are discussed below.

Although double immune staining was not conducted, which would have further elucidated alterations to CB_1_R expression within different cell types, based on morphology and anatomic distribution, CB_1_R expression appears to be primarily neuronal. Because the gp120-tg mouse model utilized is under a GFAP promotor, uniquely found in astrocytes, the increase in presumptively neuronal CB_1_R is likely induced by inflammatory molecules from glial cells. Cytokines are capable of increasing CB_1_R expression (Jean-Gilles et al. 2015). In this study cytokine activation of CB_1_R would likely be astroglia-derived rather than microglia-derived because GFAP expression, indicative of increased astroglia activation, was significantly increased, with a large effect size, in gp120-tg mice; whereas IBA1 expression, indicative of microglial activation, was not significantly increased in gp120 and only demonstrated a small to moderate effect size. Alternatively, astroglia-secreted gp120 may have a direct effect on the endocannabinoid system. For example, gp120 has been shown to activate fatty acid amide hydrolase (FAAH), an enzyme responsible for the breakdown of the endogenous endocannabinoids, anandamide and 2-arachidonoylglycerol, decreasing endocannabinoid levels (Bari et al. 2010). In addition to gp120, other HIV proteins detectable in PWH on ART, such as Tat, directly affect the endocannabinoid system. Tat impairs CB_1_R receptor function in vitro primary rat HC neurons (Wu and Thayer 2020). It is possible that in this study gp120 is decreasing endogenous cannabinoids or impairing CB_1_R receptor function, which could lead to a homeostatic upregulation of CB_1_R receptors. It is unclear why there was a significant increase in CB_1_R in the hippocampus but only a small increase in the frontal cortex of gp120 mice despite increased GFAP in both areas, however, within different brain regions CB_1_R have different properties and sub-compartmental localizations. For example, CB_1_R receptors are evenly distributed between glutamatergic and GABAergic neurons within the frontal cortex but within the hippocampus are predominately localized to GABAergic neurons (Saumell-Esnaola et al. 2021). Additionally, post-mortem analyses of the frontal cortices of PWH demonstrated decreased neuronal CB_1_R but increased astroglia CB_1_R in HAND brains (Swinton et al. 2021). Therefore, it is possible that the staining method utilized in the current study was not sensitive enough to identify subtle changes amongst different cell types. Future studies utilizing double immunolabelling are needed.

Concerningly, ART drugs themselves have been implicated in a variety of pathologies associated with HAND, including neurotoxicity, neuroinflammation, microglia activation, and metabolic dysfunction (Fields and Ellis 2019; Fields et al. 2019; Gaskill et al. 2021; Tripathi et al. 2020). One such drug, tenofovir disoproxil fumarate (TDF), a nucleoside reverse transcriptase inhibitor used in ART combinational therapies and pre-exposure prophylaxis (PrEP) has been shown to decrease neurogenesis, induce peripheral neuropathy, induce astrogliosis, and alter mitochondrial function (Fields et al. 2019; Xu et al. 2017). Since the development of ART PWH are living longer and individuals on PrEP can also experience years to decades of ART exposure. Therefore, in addition to elucidating the pathophysiology of HAND, it is also imperative that the long-term effects ART drugs have on brain health be determined.

Interestingly, TAF had a differential effect on CB_1_R in gp120-tg and wt mice, increasing expression in wt mice, but attenuating increases in gp120-tg mice. Given the evidence for mitochondrial alterations in the gp120-tg mouse model (Avdoshina et al. 2016; Fields et al. 2015; Fields et al. 2019; Swinton et al. 2019), these differential effects of TAF on the ECS may reflect varied levels of interactions between the mitochondrial biogenesis and mitochondrial fission/fusion processes and mitochondrial CB_1_R in neurons. Because CB_2_R receptors are primarily localized to microglia and involved in immune function, future studies should also investigate CB_2_R. Because there are not adequate murine anti- CB_2_R antibodies for use in IHC, future studies would need to use alternative methods of analysis such as RT-PCR or the use of antagonists/agonists to study downstream pathwasy. However, it should be noted that based on post-mortem analysis, although both CB_1_R and CB_2_R expression are altered in HAND brains and CB_2_R expression decreases with age (Swinton et al. 2021), therefore CB_2_R investigations may be more beneficial at early time points. Additionally, studies to elucidate the effect gp120 and TAF have on CB_1_R activity and downstream molecular pathways are needed.

### Effects on Locomotor Activity

Spontaneous locomotor activity testing is often used to assess general animal well-being and their ability and comfort to move freely within a familiar environment. The animal’s performance can be affected by motor function, pain, and stress (Kobayashi et al. 2020). Overall, gp120 did not have a significant effect on activity (Fig. 4, Table 2). However, TAF did have a medium to large effect on locomotor activity measures, including decreasing total distance traveled in both gp120 and wild-type mice. This could be due to a number of reasons including stress, pain, or sensorimotor deficits. Previous studies indicate that TDF induces peripheral neuropathy and hyperalgesia in both gp120 and wild-type mice (Fields et al. 2019). It is possible that TAF is having the same effect, however, future nerve conduction and pain threshold studies would be needed. TAF also lead to a decrease in rearing activity which was also seen in gp120 mice but to a lesser degree. In what manner rearing activity relates to anxiety is controversial with some proposing that increased rearing is a sign of anxiety and others that decreased rearing is a sign of anxiety because rearing represents a normal exploratory behavior (Seibenhener and Wooten 2015). This question is further complicated by the existence of different rearing types as well as sex differences (Sturman et al. 2018). Although direct conclusions about anxiety cannot be made from the non-specific locomotor activity measured in this study, it is interesting that gp120 and TAF altered potential anxiety-related activity and that on a cellular level gp120 and TAF also induced the changes in CB_1_R (Fig. 1, Table 1), a receptor known to be involved in the neurobiology of anxiety (Petrie et al. 2021). In a broader sense, CB_1_R antagonism has been shown to reduce locomotor, exploratory, and rearing activity (Bogathy et al. 2019). Further studies using tests more sensitive to anxiety-related behaviors are needed. Behavioral studies evaluating the effects of gp120 and TAF on cognition, learning, and memory are also needed. Not only are these central deficits in HAND, but CB_1_R is highly expressed in the hippocampus and has a role in short-term memory, long-term episodic memory, working memory, and recognition memory (Robledo-Menendez et al. 2022).

### IBA1

Previous studies have shown robust microgliosis in gp120 mice which is attenuated by TDF. The same pattern is demonstrated here, but with a smaller effect (Fields et al. 2019). It is unclear why this discrepancy exists in this cohort, but it is possibly due to higher levels of baseline microgliosis in wild-type vehicle-treated animals. Additionally, more significant changes in IBA1 can be seen with fluorescent secondaries compared to NovaRED-HRP (Fields et al. 2019) which was the staining method used in this study (Table 3).

This study has several limitations that are noteworthy and may guide future investigations. Despite testing several antibodies, IHC for CB_2_R or other endocannabinoid components were not performed due to a lack of antibodies able to produce a reliable IHC signal in wt and gp120-tg mice. The limited amount of brain tissues available precluded more in-depth analyses on previously reported pathways. The gp120-tg mouse does not involve viral replication and therefore is not exactly reflective of the brain in PWH on suppressive ART. It will be important to perform similar analyses on the EcoHIV or HIV+ humanized mice on suppressive ART. More behavioral tests focusing on learning and memory would provide more insight into the relevance to the gain population. Future studies utilizing more aged mice (15-18 months) would likely be more reflective of the aging population of PWH and may also reproduce the previously reported increases in IBA1 in the gp120-tg mice (Table 4).

In conclusion, given the mounting evidence that cannabis use may be neuroprotective in the context of HIV and the modulations of the endocannabinoid system, these findings support a role for the endocannabinoid system in HIV- and ART-induced neurological dysfunction. Moreover, TAF, as compared to TDF, may have a reduced effect on astroglial function and be a safer alternative to TDF for PWH. More studies are needed to better understand the molecular effects of TAF on the ECS, inflammation, and neurodegeneration.

## Data Availability

All data will be made available upon reasonable request.
