# Correction: GP120 and tenofovir alafenamide alter cannabinoid receptor 1 expression in hippocampus of mice

**DOI:** 10.1007/s13365-023-01192-6

**Published:** 2024-01-04

**Authors:** Jacqueline Renee Kulbe, Alexandra Anh Le, Michael Mante, Jazmin Florio, Anna Elizabeth Laird, Mary K. Swinton, Robert A. Rissman, Jerel Adam Fields

**Affiliations:** 1 University of California, San Diego Department of Neurosciences, San Diego, CA USA; 2 Department of Physiology and Neuroscience, Keck School of Medicine of USC, Alzheimer’s Therapeutic Research Institute, San Diego, CA USA

**Correction to: Journal of NeuroVirology (2023) 29:564-576** 10.1007/s13365-023-01155-x

There are errors in Fig. 1 of the original article. The corrected Fig. 1 follows:

**Fig. 1:**
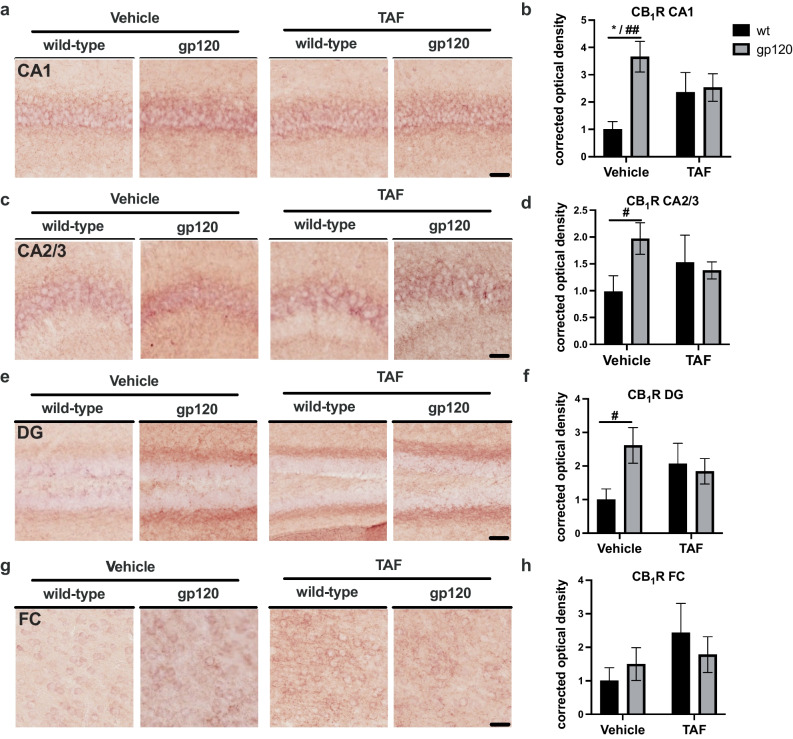
GP120 increases CB_1_R expression in mouse hippocampi, which is reversed by TAF treatment** a **CB_1_R immunostaining of CA1. **b** Quantification of corrected optical density for CA1. **c** CB_1_R immunostaining of CA2/3. **d** Quantification of corrected optical density for CA2/3. **e** CB_1_R immunostaining of DG. **f** Quantification of corrected optical density for DG. **g** CB_1_R immunostaining of FC. **h** Quantification of corrected optical density for FC. Analyzed with two-way ANOVA (*) and T-tests (#): *p,0.05, ^#^*p* < 0.05, ^##^*p* < 0.01

